# Sources and Level of Rare Earth Element Contamination of Atmospheric Dust in Nigeria

**DOI:** 10.5696/2156-9614-11.30.210611

**Published:** 2021-06-17

**Authors:** Tesleem O. Kolawole, Omowunmi S. Olatunji, Olumuyiwa M. Ajibade, Charles A. Oyelami

**Affiliations:** 1 Department of Geological Sciences, Osun State University, Osogbo, Osun State Nigeria.; 2 Department of Geology and Mineral Sciences, Crawford University, Igbesa, Ogun State Nigeria.; 3 Department of Earth Sciences, Olabisi Ona-Banjo University, Ago-Iwoye, Ogun State, Nigeria.

**Keywords:** atmospheric dust, rare earth element, fluid catalytic cracker, vehicular exhaust, upper continental crust

## Abstract

**Background.:**

Rare earth element (REE) composition of atmospheric dust has recently been used to trace potential sources of dust pollution.

**Objective.:**

The present study aimed to determine the sources of atmospheric pollution in the study area using REE patterns and determine their level of pollution.

**Methods.:**

Twenty-five (25) atmospheric dust samples were collected in the study area, with five samples each from an industrial area, traffic area, dumpsite area, residential area and remote area in Ibadan, southwestern Nigeria. In addition, five (5) topsoil and two (2) rock samples (granite gneiss) were collected for comparison. Concentrations of REE were determined by inductively coupled plasma mass spectrometry (ICP-MS).

**Results.:**

The ratio of lanthanum/cerium (La/Ce), especially in some locations in industrial area (1.5), traffic area (1.5) and to some extent dumpsite area (1.1) was higher than in soil (0.2), upper continental crust (0.5) and the minimum value of fluid catalytic crackers (1.0). Generally, the respective average values of the ratios of La/praseodymium (Pr), La/neodymium (Nd) and La/samarium (Sm) in industrial area (32.1, 7.8 and 52.6) and traffic area (14.9, 4.4 and 26.8) were higher than their respective averages in soil (4.4, 1.1 and 6.2), rock (5.7, 1.9 and 14.1), upper continental crust (4.4, 1.1 and 6.6) and the minimum value in fluid catalytic crackers (5.8, 3.7 and 37.0). Meanwhile, their corresponding value in the dumpsite area, residential area and remote area were lower or similar to the geological background levels.

**Discussion.:**

The contamination factors of REEs in the atmospheric dust of the industrial area and traffic area were classified as heavily contaminated, especially with light lanthanoid elements in REE. The degree of contamination of REEs in the atmospheric dust of industrial area (30.9) and traffic area (18.8) fell within the considerable contamination category. The high values of the light lanthanoid ratio and the contamination indices were attributed to their emission from the fired-power plant and vehicular exhaust.

**Conclusions.:**

Most of the composition of the atmospheric dust was sourced from the local geology of the study area as observed in the residential area and remote area, while the contamination in the industrial area and traffic area was attributed to human activities.

**Competing Interests.:**

The authors declare no competing financial interests.

## Introduction

Airborne dust has been linked to many adverse health effects, influences on the local and regional climate, transfer of nutrient elements to the biosphere and dispersal of anthropogenic contaminants.^1^–^5^

Most airborne dusts are generated from weathered products of both local and regional geology, which are subsequently transported by wind. Atmospheric dust is often affected by surface and photochemical processes and mixed with air masses that contain other types of aerosols including the products of biomass burning and other anthropogenic materials.^6^

Trace metal composition of atmospheric dust has been useful in providing clues to the origin of dust and its harmful effects.^7^ However, recently, rare earth element (REE) concentrations of atmospheric dusts have been used in investigations to trace the potential sources of environmentally deleterious materials. These REEs are described in Table 1 on the basis of their molecular weight, application and possible health effects. Rare earth elements are known to occur naturally as accessories in rocks and minerals^8^ and in some types of deposits such as alkaline igneous deposits,^9^–^10^ coal deposits,^11^ and heavy mineral placer deposits.^12^ The estimated average concentration of REEs in the Earth's crust ranges from 130 μg/g to 240 μg/g, which is significantly higher than other commonly exploited elements, and much higher than the respective chondrite abundances.^13^ They are mined from those deposits and are being utilized in modern industries for the production of numerous new materials such as electronics (television screens, light emitting diode (LED) bulbs), technology (lasers, optics glass, nuclear batteries), and the medical sciences (portable X-ray machine, X-ray tube).^14^ Indiscriminate dumping and incineration of e-waste is facilitating the significant release of REEs and other toxic elements to soil, water and air.

**Table 1:** Description of Rare Earth Elements^18^–^19^

**Chemical symbol**	**Name**	**Molecular weight**	**Application**	**Possible health effects**
La	Lanthanum	138.905	Petroleum refining catalytic converters, fuel additives, chemical processing, metal alloys, ceramics	Cytotoxicity to lung tissue, Pneumoconiosis
Ce	Cerium	140.116	Petroleum refining catalytic converters, fuel additives, chemical processing, metal alloys, ceramics	Pneumoconiosis
Pr	Praseodymium	140.908	Petroleum refining catalytic converters, fuel additives, chemical processing, metal alloys, ceramics, computer hardware drive, antilock brakes	Pneumoconiosis
Nd	Neodymium	144.243	Petroleum refining catalytic converters, fuel additives, chemical processing, metal alloys, ceramics, magnetic (computer hardware drive, antilock brakes), electrical appliances (LCD TV) medical imaging, laser fiber optics, satellite communication	Pneumoconiosis, cardiovascular diseases (arteriosclerosis), cerebral cortex
Pm	Promethium	144.913	Light source for signals in a heat button, research purpose	-
Sm	Samarium	150.360	Satellite communication, aircraft structure	
Eu	Europium	151.964	Ceramics, electrical appliances (LCD TV) medical imaging, laser fiber optics	
Gd	Gadolinium	157.25	Ceramics, glass and polishing, Electrical appliances (LCD TV) medical imaging, laser fiber optics	Renal toxicity (Nephrogenic system fibrosis)
Tb	Terbium	158.925	Computer hardware drive, antilock brakes, electrical appliances (LCD TV) medical imaging, laser fiber optics	
Dy	Dysprosium	162.500	Computer hardware drive, antilock brakes	
Ho	Holmium	164.930	Glass and polishing	
Er	Erbium	167.259	Electrical appliances (LCD TV) medical imaging, laser fiber optics	
Tm	Thulium	168.934	-	
Yb	Ytterbium	173.055	Ceramics	
Lu	Lutetium	174.967	Ceramics	

In addition, REEs are used in fluid catalytic cracking, which is one of the most important conversion processes used in petroleum refineries. It is used to convert the high-boiling point, high-molecular weight hydrocarbon fractions of petroleum crude oils into more valuable light-weight hydrocarbons such as gasoline and oil fuel.^15^ The fluid catalytic crackers unit primarily uses zeolite catalysts. The zeolite cracking catalyst contains mainly REE mixtures. Therefore, vehicles and power plants that utilize hydrocarbon products emit REE into the atmospheric environment. Many studies have identified the presence of REEs, especially the light lanthanoids in the atmosphere^2^,^16^–^17^ and attributed their presence to this type of emission. It was established from those studies that REEs were a substrate of modern catalytic converters in the refining of petroleum products. Therefore, REE are expected to be emitted as abrasion particles from the catalyst to the atmospheric environment. Consequently, REE distribution of airborne particulate matter helps in identifying pollution events and can be used as a tracer for different atmospheric hydrocarbon emissions, especially in an urban area such as Ibadan, Nigeria.^20^

**Abbreviations**
*Cf*: Contamination factor
*Cd*: Degree of contamination
*HREE*: Heavy rare earth element
*LREE*: Light rare earth element
*MREE*: Middle rare earth element
*PI*: Pollution index
*REE*: Rare earth element
*ΣREE*: Total rare earth element

Ibadan is one of the largest cities in Nigeria in terms of land mass with a total area of 3080 km^2^ and population of more than 3.57 million people.^21^ Within the city, there are a number of anthropogenic activities that could cause air pollution. These include mineral dust re-suspension by wind, vehicular emissions, quarry activities, industrial emissions, indiscriminate burning of domestic waste in illegal dumps, and traffic congestion. Investigation of sources of pollutants in atmospheric dust in Nigeria at large and in the study area in particular have been primarily focused on trace element distribution and characterization.^22^–^29^ The major findings from their studies traced the different potentially toxic metals (trace elements) to sources such as anthropogenic (industrial and vehicular activities) and geogenic (sea salt, soil dust re-suspension) activities. However, despite recent advancements in tracing the source of atmospheric pollutants using REE^15^,^30^–^31^,^17^,^32^–^34^ and knowledge of the harmful effects of REE on human health,^35^–^37^ to the best of our knowledge there have been no previous studies on this topic in Nigeria as a whole or in the study area. Therefore, the present study investigated the composition and distribution of REE in the atmospheric dust of selected areas in Ibadan city, in order to determine sources of pollution.

## Methods

**Figure 1:**
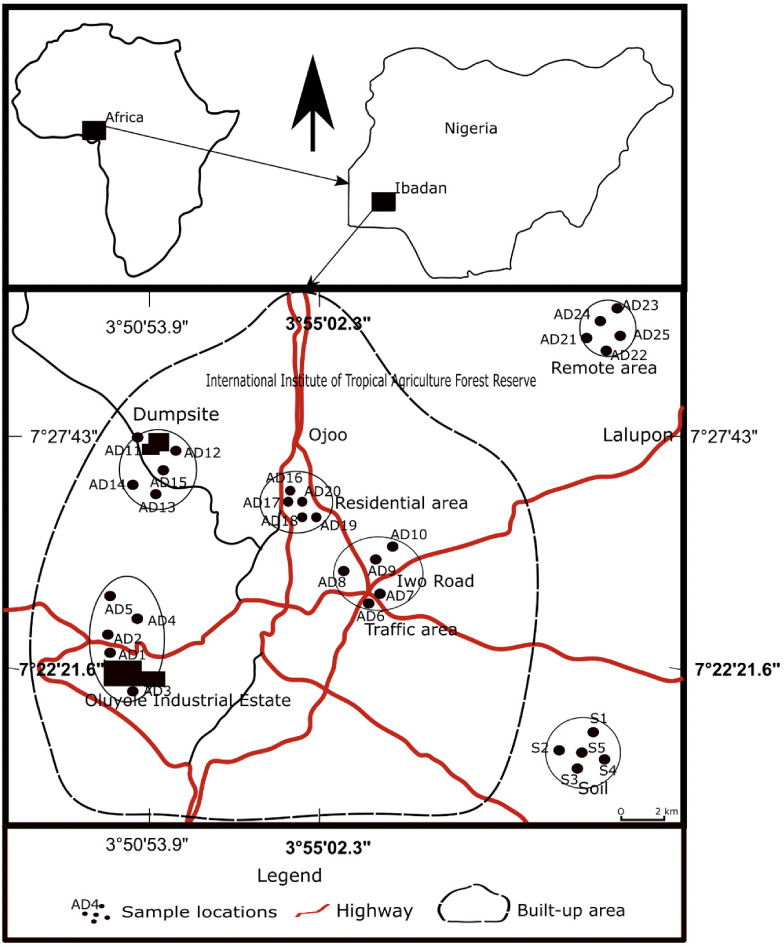
Map of the study area with sampling points

Ibadan is located in the southwestern part of Nigeria, about 143 km northeast of Lagos. The city is on the railroad line linking Lagos with the northern part of the country and is well connected by road to other cities in the region. The study was located within Ibadan Urban Center and the suburb *(Figure 1).* Four zones based on land use patterns (urban units) within Ibadan were identified and selected for sampling purposes. These zones reflect the major types of human activities such as industrial activities, traffic, and open burning of solid waste in landfills that could cause pollution of the environment in the study area. The identified land use zones included a residential area, industrial area, dumpsite area, and traffic area. Roads in Ibadan can be classified as major expressway (main), sub-major, and minor roads. Most of these roads are poorly maintained and some are unpaved. An increase in vehicle traffic due to increasing vehicle ownership has led to traffic congestion. For the past two decades, Ibadan city has been gradually developing into an important industrial city in Nigeria, but this increasing industrialization has not been matched with proper planning to prevent or mitigate associated environmental pollution problems. Most industries are concentrated in the southwestern part of the city (Oluyole Industrial Estate). The present study monitors atmospheric dust in Oluyole industrial area which accommodates various industries ranging from small-scale to large-scale. These include a bottling company, diaper manufacturer, and industrial bakery, most of which utilize oil-fired power plants, generating airborne particles associated with possible health impacts. A permitted solid waste landfill is located at Awotan in Ibadan (*Figure 1*). However, unlike practices in well-developed solid waste landfills, the dumping of refuse in this facility is usually done indiscriminately without any prior sorting. Some of the activities that generate particulate matter and disperse it in air around and within the waste facilities include movement of waste to and from the facility, off-loading and compaction of waste, incineration of waste, and wind scouring of waste surfaces.

The study area lies within Ibadan, southwestern Nigeria and the dominant rock types are quartzites, banded gneisses, augen gneisses and migmatites.^38^–^40^ These rocks were intruded by pegmatite, quartz vein, aplite and dioritic dykes. Other minor rocks within the area included amphibolites and pegmatites. In many locations within the study area, the rocks are overlain by thick weathered regolith with few outcrops. Ibadan is located within the forest grassland of southwestern Nigeria and lies in the transition zone between the humid and the sub-humid tropical climates. The climate is characterized by well-defined rainy (wet) and dry seasons. There are two pronounced rainy seasons: April–June and September–November. There is a minor dry season between July and August which divides the wet season into two, while the major dry season is from December–February, when the northeasterly wind known as the Harmattan wind blows from the Sahara region. The mean annual rainfall ranges from 788 mm–1844 m, with a mean temperature of 26°C. The monthly mean temperature is between 25°C and 30°C throughout the year.

### Sample collection

Atmospheric dust samples were collected in four different urban units within Ibadan city: the industrial area (located at Oluyole Industrial Estate and Nigeria Breweries); traffic area (Ojoo, Iwo Road, Sango); dumpsite area (Awotan); and residential area (Agbowo) (*Figure 1*). Background samples were collected in a remote area where anthropogenic activities are absent or minimal, but reflecting the same geology as the other sampled areas. The test samples were collected from four (4) two-floor buildings in each urban unit. The buildings were selected randomly based on accessibility to the building and were located a minimum of 50 m and a maximum 750 m from the hotspots. The sample collection was done during the lull of the raining season to prevent atmospheric transportation from long distances. The sampling was carried out using the dry deposition method with petri dishes (settling plates) mounted on the rooftops of the building in each location for 72 hours. Twenty-five (25) atmospheric dust (AD) samples were collected, with five samples collected from each location (AD1-25) Five topsoil samples were collected at a depth of 5 cm in a pristine environment (S1-5) and two of the most dominant rock samples (granite gneiss) in the study area were collected, in order to establish natural background values and the anthropogenic contribution of the deposited dust.

### Sample analysis

Both the atmospheric dust and soil samples were sieved through <63-μm mesh to remove large pieces of organic matter such as leaves, insects, and very coarse particles. The REE concentration of the digested samples was determined with inductively coupled plasma mass spectrometry and was conducted by Activation Laboratory in Canada. Five grams (5 g) of each of the samples were digested using aqua regia solution, which involved adding 5 ml of nitric acid (Merck Suprapur 65%), 2 ml of hydrochloric acid (Merck Suprapur 36%) and 10 ml of ultra-pure water in a Pyrex tube. The mixture was heated for 2 hours at 95°C on a hot plate. The extracted solution was filtered with a Whatman n°41 (WH1441-110) filter, diluted to 50 ml with ultra-pure water and kept in pre-cleaned polyethylene bottles in the refrigerator until analysis. Quality assurance and quality control procedures were conducted by using standard reference materials: United States Geological Survey's Geochemical Exploration Reference Samples (GXR)-1, GXR-2, GXR-4 and GXR-6. Recoveries of REE were between 93%–101%.

## Results

Rare earth elements are usually divided into three groups: light REE (LREE) from La to Nd (138.905 to 144.243 molecular weight), middle REE (MREE) from Sm to Dy (150.36 to 162.5 molecular weight), and heavy REE (HREE) from Ho-Lu (164.93 to 174.967 molecular weight). The light REE are found in higher amounts in the environment.^41^ Note that promethium (Pm), in the category of LREE, was not analyzed, because it is extremely scarce and the equipment used (ICPMS) for the analysis could not detect it.

The statistical summary of REE concentrations in atmospheric dust across areas affected by human activity (industrial, traffic, dumpsite and residential areas) is presented in Table 2, while that of local soil and existing rock of the study area is presented in Table 3. The highest mean concentrations of cerium (Ce) (330.80 mg g^−1^); dysprosium (Dy) (2.12 mg g^−1^), erbium (Er), (0.89 mg g^−1^); europium (Eu), (0.86 mg g^−1^); gadolinium (Gd), (4.75 mg g^−1^); lanthanum (La) (251.26 mg g^−1^); neodymium (Nd), (32.47 mg g^−1^); praseodymium (Pr), (8.19 mg g^−1^); samarium (Sm), (4.94 mg g^−1^); and terbium (Tb), (0.50 mg g^−1^) in atmospheric dust were observed in the industrial area, and concentrations of holmium (Ho), (0.36 mg g^−1^); lutetium (Lu), (0.14 mg g^−1^), and ytterbium (Yb), (0.89 mg g^−1^) were recorded in atmospheric dust in the traffic area. On the basis of the mean concentrations, REEs generally decreased in the order of Ce>La>Nd>Pr>Sm>Gd>Dy>Er>Yb> Eu>Tb>Ho>Tm>Lu. This concentration sequence was also seen in the local soil and rock of the study area. However, a higher concentration of La was observed in some of the samples in the industrial area, traffic area and to some extent dumpsite area. Furthermore, the calculated total REE (ΣREE) contents of the atmospheric dust varied from one urban unit to another. The values ranged from 42.45 mg g^−1^ (residential area) to 784.49 mg g^−1^ (industrial area), the ΣREE of soil varied from 108.95–159.37 mg g^−1^ *(Table 1)* and the average of ΣREE of rock was 103.10 mg g^−1^. The average ΣREE values in the industrial, traffic, dumpsite, residential, and remote areas and local soil were 638.26, 282.57, 129.64, 162.77, 95.99 and 143.59 mg g^−1^, respectively. The average ΣREE in dusts in the industrial area and traffic area was about 4.5- and 2.0-fold higher than the average ΣREE of the soil, upper continental crust, and rocks, respectively, while the ΣREE in the atmospheric dust of the dumpsite and residential area were similar to those in soil, upper continental crust and rocks and higher than the remote area. In addition, in an effort to determine whether the differences in concentrations of REE observed across zones are statistically significant, a further analysis of the data was carried out using the analysis of variance (ANOVA) method *(Table 3).* There was no significant difference (P>0.05) between concentrations of REE across environmental zones. However, there was a marked significant difference (P<0.05) in light REE (La, Ce, Pr and Nd), and this was further determined by post hoc testing for multiple comparison of ANOVA.

**Table 2:** Summary of Rare Earth Elements in Atmospheric Dust within Areas Affected by Human Activity

**REE**	**Industrial (N=5)**		**Traffic (N=5)**		**Residential (N=5)**		**Dumpsite (N=5)**	
	Mean±Std	Range	Mean±Std	Range	Mean±Std	Range	Mean±Std	Range
La	251.26±127.20	113.44–430.87	114.75±111.15	19.14–253.95	23.78±18.11	18.40–30.44	21.69±5.27	9.86–30.44
Ce	330.80±139.45	291.47–378.32	126.19±57.52	49.64–176.87	64.60±13.22	44.34–78.83	106.95±37.11	35.36–356.11
Pr	8.19±1.02	7.02–9.53	6.57±1.94	4.63–9.17	6.55±1.07	5.61–7.98	3.71±1.85	2.26–6.85
Nd	32.47±13.90	20.39–52.40	22.67±5.78	16.91–30.55	22.93±3.91	19.14–28.10	13.21±6.35	8.28–24.02
Sm	4.94±0.56	4.38–5.73	3.82±0.80	3.04–4.92	4.13±0.71	3.51–5.07	2.38±0.99	1.55–4.02
Eu	0.86±0.15	0.71–1.10	0.48±0.03	0.44–0.51	0.44±0.07	0.34–0.54	0.40±0.20	0.19–0.73
Gd	4.75±0.83	3.92–6.00	3.37±0.68	2.51–4.30	3.27±0.43	2.77–3.84	2.28±1.38	1.41–4.70
Tb	0.50±0.08	0.39–0.59	0.39±0.08	0.31–0.49	0.39±0.04	0.34–0.44	0.24±0.10	0.18–0.41
Dy	2.12±0.32	1.66–2.48	1.91 ±0.46	1.49–2.56	1.73±0.16	1.47–1.91	1.20±0.44	0.91–1.92
Ho	0.35±0.06	0.29–0.44	0.36±0.10	0.28–0.50	0.30±0.04	0.24–0.35	0.22±0.09	0.13–0.37
Er	0.87±0.13	0.68–1.01	0.89±0.31	0.59–1.30	0.65±0.12	0.50–0.82	0.52±0.25	0.26–0.91
Tm	0.18±0.03	0.14–0.21	0.16±0.03	0.14–0.20	0.15±0.02	0.13–0.18	0.14±0.01	0.14–0.14
Yb	0.82±0.12	0.64–0.96	0.89±0.36	0.47–1.34	0.58±0.15	0.41–0.78	0.53±0.25	0.22–0.90
Lu	0.14±0.02	0.11–0.17	0.14±0.02	0.11–0.17	0.13±0.01	0.11–0.14	0.13±0.02	0.11–0.14
*ΣREE*	*638.26*	*532.92–784.49*	*282.57*	*100.56–482.86*	*129.64*	*98.9–146.83*	*162.77*	*77.80–427.39*

Abbreviations: REE, rare earth element

**Table 3:** Summary of Rare Earth Elements in Atmospheric Dust of Remote Area, Local Soils, and Rocks of the Study Area

**REE**	**Remote (N=5)**		**Soil (N=5)**		**Rock**	**Average UCC^42^**	**C-F**	**p-Value**
	Mean	Range	Mean	Range	Average (N=2)			
La	12.71±6.94	7.34–23.05	19.44±3.15	14.18–22.40	29.2	30.00	8.13	0.01*
Ce	63.58±48.94	21.61–147.37	89.99±13.12	68.62–100.97	49.4	60.00	10.45	0.01*
Pr	2.95±1.50	1.82–5.18	4.41±0.75	3.17–5.10	4.7	8.20	8.81	0.13
Nd	10.35±5.12	6.47–17.94	17.54±2.79	12.89–19.90	14.3	28.00	5.49	0.01*
Sm	1.90±0.87	1.15–3.13	3.00±0.42	2.30–3.30	2.1	6.00	11.36	0.40
Eu	0.31±0.10	0.20–0.42	0.52±0.10	0.36–0.60	0.4	1.20	10.14	0.26
Gd	1.69±0.56	0.99–2.45	2.98±0.45	2.23–3.40	1.4	5.40	8.93	0.17
Tb	0.20±0.08	0.12–0.32	0.37±0.05	0.29–0.40	0.1	0.90	13.43	0.24
Dy	0.97±0.44	0.55–1.60	2.35±0.29	1.94–2.70	0.7	3.00	12.12	0.18
Ho	0.18±0.08	0.10–0.30	0.40±0.06	0.35–0.50	0.1	1.20	8.42	0.10
Er	0.44±0.20	0.23–0.70	1.15±0.15	1.01–1.40	0.2	2.80	9.36	0.06
Tm	0.13±0.03	0.07–0.14	0.13±0.05	0.09–0.20	0.1	0.48	3.02	0.02*
Yb	0.46±0.21	0.24–0.71	1.12±0.17	0.97–1.40	0.2	3.00	6.96	0.08
Lu	0.13±0.03	0.07–0.14	0.18±0.03	0.14–0.20	0.1	0.50	5.06	0.01*
*ΣREE*	*95.99*	*42.45–167.57*	*143.59*	*108.655–159.37*	*103.1*	*150.70*	-	-

Abbreviations: C-F, calculated F-Value; REE, rare earth element; UCC, upper continental crust.

### Chondrite-normalized rare earth element pattern

**Figure 2:**
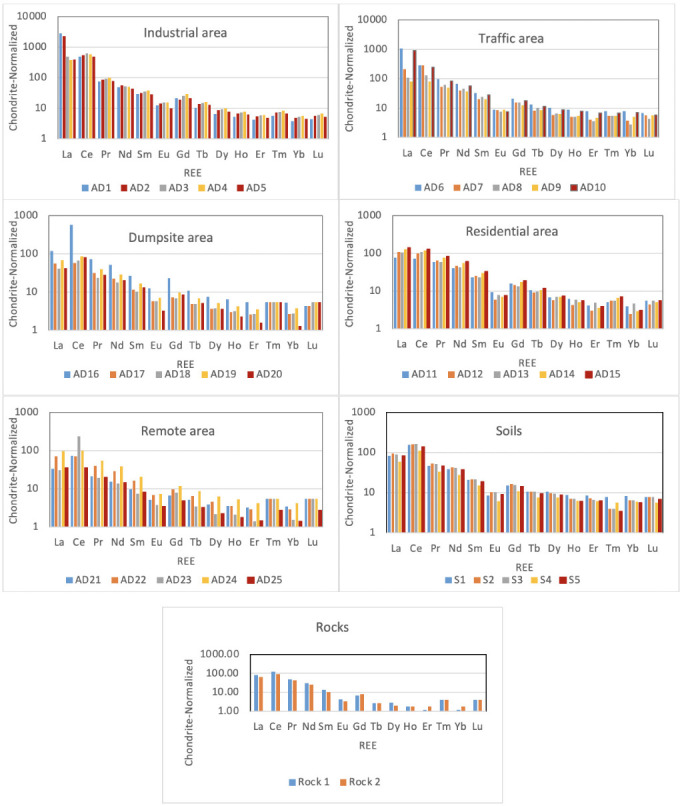
Chondrite-normalized rare earth element (REE) patterns for atmospheric dusts across zones in the study area

Rare earth elements were selected on the basis of their broadly similar chemistry and changes in their relative concentrations are easier to interpret than the changes in 14 randomly selected elements. It is therefore important to normalize to their initial condition, and chondrite is often used for this normalization. This will help to decipher the enrichment or depletion of REE and to determine if the enrichment or depletion is a result of geogenic or anthropogenic contribution. Therefore, chondrite-normalized REE patterns^42^ were used to determine the relative changes of REEs in the studied atmospheric dusts, soil, and rock. The variation in REE composition of the atmospheric dust across different environmental units is presented in Figure 2. Most of the atmospheric dust samples exhibit similar patterns and there were no drastic changes in the REE pattern of the dust. The general trend of the REE patterns is such that there is enrichment of Ce followed by sharp inclination of middle REE (Sm-Dy) to light REE (La-Nd). The trend also showed the depletion of Eu, and a generally gently inclined slope for the other middle REE to heavy REEs (Ho-Lu). Furthermore, these patterns were all similar to the chondrite-normalized pattern found in the soil and rock of the study area. Meanwhile, there was slight deviation in La in some of the samples that were very close to the industrial area (AD1 and AD2) and traffic area (AD6 and AD10) and to some extent the dumpsite. The LREE/HREE values of atmospheric dust ranged from 37.78–45.22 (industrial area), 14.16–33.27 (traffic area), 17.04–23.57 (dumpsite), 16.28–44.43 (residential area), 15.21–51.44 (remote area) and 14.24–16.90 (soil) with average values of 42.18, 23.10, 19.67, 22.77, 23.48 and 15.52, respectively.

### Rare earth element fractionation

**Figure 3:**
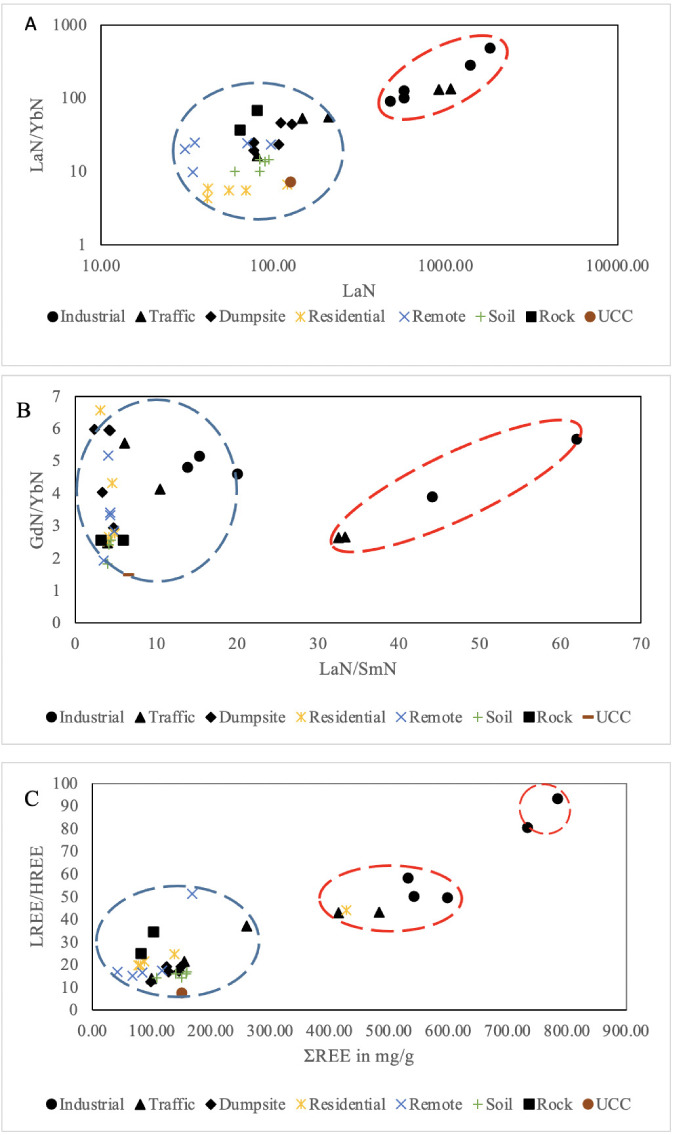
Total rare earth element fractionation pattern diagram of atmospheric dust, local soil, rock and upper continental crust in the study area by; (A) La_N_ vs. La_N_/Yb_N_; (B) La_N_/Sm_N_ vs Gd_N_/Yb_N_; (C) Bivariate plot of relationship between total REE (ΣREE) and LREE/HREE in the study area

The established chondrite-normalized ratio of some randomly selected REEs such as La_N_ vs La_N_/Yb_N_ and La_N_/Sm_N_ vs Gd_N_/Yb_N_ have also been successfully used to determine the provenance of dust.^43^,^17^ Fractionation of REEs are established standards used to further determine the provenance of REE. Figure 3A showed the REE fractionation pattern of atmospheric dust in different zones, local soil, rock, and upper continental crust by La_N_ vs La_N_/Yb_N_. It was observed that a close variation exists in La_N_ values of the local soil (59.85) and rock (68.61), while wide variation was observed between upper continental crust (126.58) and local geologic materials. Likewise, there was intermediate variation in the ratio of La_N_ and Yb_N_ of upper continental crust (7.17) and local soil (14.61), and a wide variation between this ratio and rock (80.72) in the study area. Meanwhile, the atmospheric dust of the residential area, dumpsite area, remote area and to some extent traffic area fell within the range of the values of La_N_ and La_N_/Yb_N_ for geologic materials. However, the atmospheric dust in the industrial area and few of the traffic area samples had higher La_N_ (478.68–1818.00) and La_N_/Yb_N_ (91.42–482.90) values compared to their corresponding values in the geological materials and other atmospheric dust.

In addition, the analysis of La_N_/Sm_N_ versus Gd_N_/Yb_N_ (*Figure 3B*) of the atmospheric dust and the previously mentioned geologic materials found that La_N_/Sm_N_ values showed a close relationship between the atmospheric dust of residential area, dumpsite area and remote area (2.34–5.56), and geologic materials (3.22–6.56) (local soil, rock of the study area and upper continental crust), while the corresponding values of Gd_N_/Yb_N_ was somewhat higher in atmospheric dust (1.83–6.58) than the geologic materials (1.48–2.55). However, the values of the ratio of La_N_ to Sm_N_ in the atmospheric dust of industrial area and traffic area (20.10–61.95) was a comparatively higher ratio than the other atmospheric dust and all the geologic materials under consideration.

The bivariate plot of LREE/HREE and ΣREE presented in Figure 3C showed that the atmospheric dust of residential area, remote area and dumpsite area was similar to the value of both LREE/HREE (7.72–34.5) and ΣREE (25.24–169.37 mg/g) of geologic materials. In addition, there was a positively strong correlation (0.91) between LREE/HREE and ΣREE of atmospheric dust and geologic materials, which indicate their common source. However, the atmospheric dust in the industrial area and traffic area revealed a relatively higher values of LREE/HREE (42.95–93.40) and ΣREE (25.24–169.37) compared to local soil and rock of the study area and upper continental crust.

### Cerium and europium anomaly

Cerium is the most abundant REE and the Ce anomaly (Ce/Ce*), which is defined in Equation 1, is used to establish whether the REE in dust is inherited from the source rock/soil. It is usually described as being positive (Ce/Ce*>1) or negative (Ce/Ce*<1).^17^ A positive value indicates that REE are sourced from weathering of rock/soil, but a negative value indicates they may have another source (anthropogenic).^17^ Likewise, the europium (Eu) anomaly (Eu/Eu*) defined in Equation 2 is also used to determine if dust was inherited from source rock/soil through weathering.^17^ A negative Eu (Eu/Eu*<1) anomaly implies that the source was a result of the weathering of the source rock/soil, while a positive Eu (Eu/Eu*>1) anomaly may indicate anthropogenic contribution.


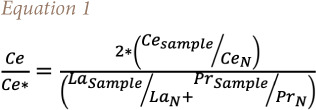



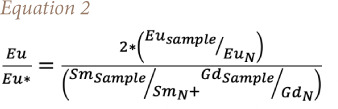


**Figure 4:**
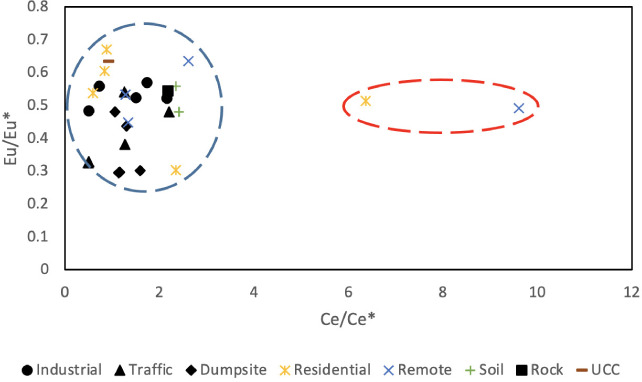
Cerium and europium anomalies in atmospheric dust across different environmental units and soils of the study area, rock, and upper continental crust

The Ce/Ce* value of atmospheric dust ranged from 0.48–2.62, most of which showed a slightly positive anomaly, except for a few which showed a negative anomaly and very few showed a strong positive anomaly (6.37–9.61). This trend was also observed in the Ce/Ce* values of geologic materials, which showed a slightly positive anomaly *(Figure 4).* Meanwhile, the Eu/Eu* values fell within the same range for both atmospheric dust in all locations (0.29–0.88) and geologic materials (0.47–0.66), which had a generally negative anomaly.

### Lanthanum to light lanthanoids ratios

Lanthanum to light lanthanoid ratios have been used successfully to distinguish anthropogenic input of REE such as from refinery emissions, oil-fired power plants, and vehicular and coal burning emissions from other sources.^2^,^16^,^17^ Table 3 presents the statistical summary of ratios of La/Ce, La/Sm, La/Nd and La/Pr of atmospheric dusts across study locations and local soils, rock of the study area, upper continental crust, and fluid catalytic crackers. It was observed that the average values of the La/Ce in the atmospheric dust of industrial area and traffic area were 4-and 1.6-fold higher than average values in local soil, upper continental crust and rock of the study area, respectively. In addition, the average value of the La/Ce in the atmospheric dust of dumpsite area was 3-fold higher than soil and slightly higher than upper continental crust and rock in the study area *(Table 3).* However, it was slightly lower than the minimum value of fluid catalytic crackers (1.0), except that a few of the maximum values of industrial area and traffic area were higher than the minimum value of fluid catalytic crackers. However, the corresponding values of La/Ce in the residential area and remote area were similar to the values of all the geologic materials. Moreover, the average values of La/Pr in the dust of the industrial area, traffic area and dumpsite area were at least 6-, 2.5- and 1.5-fold higher than the geologic materials (soil, rock and upper continental crust) and with the minimum values of fluid catalytic crackers, while the average and the maximum value of La/Pr in the dust of the residential area and remote area was similar to the values in the geologic materials. The average value of La/Nd in the atmospheric dust of the industrial area, traffic area and dumpsite area was at least 7-, 2-, and 1.2-fold higher than the local soil, upper continental crust, and rock of the study area and the minimum value of fluid catalytic crackers respectively, whereas residential area and remote area were similar to that of the geologic materials. Furthermore, the average value of La/Sm of dust in the industrial area and traffic area was 4–8 times greater than the geologic materials, while that of the dumpsite area was 2-fold higher than both local soil and upper continental crust, but less than average La/Sm of rock in the study area. However, only the average value of the industrial area and the maximum value of traffic area was higher than the minimum value of fluid catalytic crackers. Likewise, average La/Sm values of the residential area and remote area were similar to the corresponding value in the geological materials and lower than the minimum value of fluid catalytic crackers.

### Contamination factor and the degree of contamination of rare earth elements

To describe the contamination of REE in the study area, it is important to calculate the contamination factor (Cf) and degree of contamination, accordingly, using the formula developed by Hakanson, 1980, shown in Equation 3:^44^


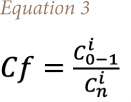


Where *Cf* is the contamination factor, *C^i^*_o–1_ is the mean value of the individual element in each site and *C^i^_n_* is the control value for the element. In the present study, the average concentration of each element in the soil was used as the control value.

The degree of contamination (C_d_) is defined as the sum of all contamination factors for a given site. The formula is given in Equation 4:

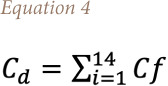



The terminology of the contamination factor and the degree of contamination is given in Table 4.

**Table 4:** Lanthanum to Light Lanthoid Ratio in Atmospheric Dust and Other Materials

**Environment**		**La/Ce**	**La/Pr**	**La/Nd**	**La/Sm**
Industrial	Mean	0.8	32.1	7.8	52.6
	Range	0.3–1.5	12.8–61.4	4.6–10.4	21.5–96.0
Traffic	Mean	0.8	14.9	4.4	26.8
	Range	0.3–1.5	4.1–27.7	1.1–8.3	6.2–51.6
Residential	Mean	0.4	3.7	1.1	5.9
	Range	0.2–0.4	2.3–4.5	0.7–1.3	3.6–7.3
Dumpsite	Mean	0.6	9	2.5	13.8
	Range	0.1–1.1	3.7–15.5	1.0–4.4	4.9–23.5
Remote	Mean	0.3	4.2	1.2	6.5
	Range	0.0–0.4	4.0–4.4	1.1–1.3	5.5–7.4
Soil	Mean	0.2	4.4	1.1	6.5
	Range	0.2–0.2	4.3–4.5	1.1–1.1	6.2–6.8
FCC^2^	Range	1.0–13.0	5.8–20.0	3.7–14.0	37.0–98.6
UCC^42^		0.5	4.4	1.1	6.6
Rock		0.56	5.77	1.93	14.05

Abbreviations: FCC, fluid catalytic cracking; UCC, upper continental crust

**Table 5:** Dust Quality Threshold for the Contamination Factor and Degree of Contamination

**Class**	**Qualification**	**Cf**	**C_d_**
1	Uncontaminated	Cf<l	C_d_<8
2	Moderate contamination	1≤ Cf<3	8≤C_d_<16
3	Considerable contamination	3≤ Cf<6	16≤C_d_<32
4	Very high contamination	Cf ≥6	C_d_≥32

**Figure 5:**
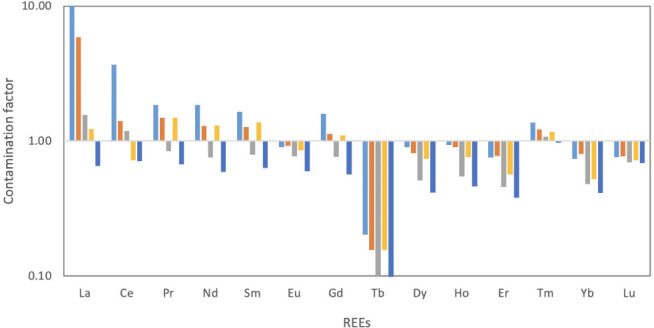
Contamination factor of rare earth elements (REEs) in the study area

**Figure 6:**
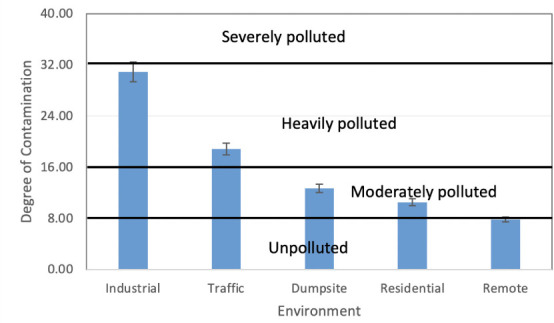
Degree of contamination of rare earth elements in the study area

The present investigation assessed the Cf of REEs in different sites and it was observed that the Cf value of La in the industrial area (12.93) and traffic area (5.91) was classified as very highly contaminated and considerably contaminated, respectively. Meanwhile, La was moderately contaminated in the dumpsite area (1.56) and the residential area (1.22) and was uncontaminated in the remote area. Generally, other light REEs (Ce-Sm) and to some extend Gd were classified within moderate contamination in the industrial area, traffic area, and dumpsite area with values ranging from 3.68 (Ce in the industrial area) to 1.10 (Gd in the residential area) and low residential area. While the values in the rural area were generally similar with the control values and termed as being uncontaminated. However, Cf values of middle and heavy REEs (Tb-Lu) in all the sites were generally within the uncontaminated class, except Tm, which was moderately contaminated *(Figure 5).* The degree of C_d_ values in the study area is such that the industrial area (30.87) and traffic area (18.84) was considerably contaminated, while the dumpsite area (12.69) and residential area (10.53) had a moderate degree of contamination. The C_d_ value in the remote area was 7.81 and fell within the uncontaminated class (*Figure 6*).

### Pollution index

The pollution index (PI) is used to assess level of pollution status of elements in a site of interest by adopting Nemerow's formula, shown in Equation 5:^45^


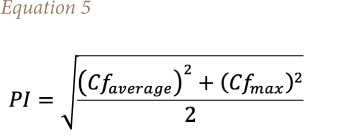


**Figure 7:**
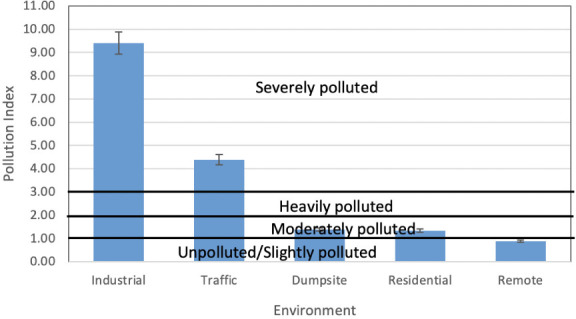
Pollution index of rare earth elements across the study area

Where *Cf_average_* the average contamination is factor and *Cf_max_* is maximum contamination factor of REEs. The interpretation of PI is such that PI<0.7 (unpolluted), 0.7<PI<1 (slightly polluted), 1<PI<2 (moderately polluted), 2<PI<3 (heavily polluted) and >3 (severely polluted). The result of calculated PI of this study is presented in Figure 7. It was observed that the industrial and traffic areas were severely polluted with a PI value of 9.41 and 4.39, respectively. The PI value of the dumpsite area was 1.39 and that of the residential area was 1.33, both indicating a moderately pollution level, while the remote area (0.88) was slightly polluted.

## Discussion

The present study showed that both the concentration of REEs (La, Ce, Pr, Nd, Sm, Eu, Gd, Tb, Dy, Ho, Er, Tm, Yb, Lu) and their ΣREE clearly varied with lower values in the remote area and to some extent in the residential area, and higher values in the industrial area, traffic area and to some extent the dumpsite area. The REEs in the dust of the residential and remote area were similar to that of the local soil, rock of the study area and average upper continental crust. Some of the atmospheric dust in the industrial, traffic, and dumpsite area were also slightly similar to the geologic materials in their REE concentrations. This indicated that most of the atmospheric dust may originate from the geology of the study area.

This pattern was also consistent with the REE chondrite-normalized pattern of the atmospheric dust, where they often showed a positive Ce anomaly and negative Eu anomaly, a composition also exhibited by the local soil and the rock of the study area. Tang and Han^18^ opined that when there is a positive Ce anomaly and negative Eu anomaly, this suggests it is the product of chemical weathering of the surface environment, and therefore, this revealed that there is an inherited relationship between atmospheric dusts and the local geology.

The above assertion was also corroborated by the REE fractionation ratios pattern of La_N_ versus La_N_/Yb_N_ and La_N_/Sm_N_ versus Gd_N_/Yb_N_, and the bivariate plot of LREE/HREE versus ΣREE. Most of the dusts, especially in the residential, dumpsite, and remote areas, clustered around the values of the geologic materials (local soil, rock of the study area and upper continental crust). This further indicated that most of the dust originated from a geogenic source. However, the import of the consistent deviation of the atmospheric dust of the industrial and traffic area from the normal cluster of geologic materials and the dust of other urban units observed in of La_N_ vs. La_N_/Yb_N_ and La_N_/Sm_N_ vs. Gd_N_/Yb_N_, and the bivariate plot of LREE/HREE vs. ΣREE was attributed to the mixing of different proportions of minerals in the basement rocks of the study area and other exogenous (anthropogenic) materials sourced from industrial and traffic-related activities. The La_N_/Yb_N_ values in the traffic, dumpsite and industrial areas were far higher than the local soil. Relatively higher values were also observed in road dust in Dhaka,^46^ urban particulate matter in Spain,^47^ and atmospheric particulate matter in Tokyo.^48^

The anthropogenic source of REEs in the study area was better assessed by La and other lanthanoid ratios such as La/Ce, La/Sm, La/Nd and La/Pr, which has been recently adopted in previous studies^2^,^16^,^18^,^45^ as pollution indicators of refinery emissions, oil-fired power plants, coal-burning and vehicular emissions. Kulkarni *et al.*^49^ and Bozlaker *et al.*^2^ reported a high La/Ce ratio in fluid catalytic crackers (1.0–13). In the present study, only industrial, traffic, and dumpsite areas had some samples that had a La/Ce ratio that ranged from 1.1–1.5, implying a slight deviation from crustal origin (Ce-rich) toward a refinery catalyst (La-rich) signature. The La/Pr and La/Nd ratios measured in this study were higher in the industrial and traffic area and to some extent the dumpsite area than fluid catalytic crackers. Kurkarni *et al.*^46^ also attributed the high ratios of La/Pr and La/Nd to vehicular emissions in Houston, Texas. In addition, a high La/Sm in industrial area (average of 52.6) and traffic area (26.8) was attributed to an oil-fired power plant in the study area. This assertion was supported by a study by Olmez and Gordon^50^ who reported an average value of 25 and 28 for the La/Sm ratio and attributed this to a refinery and oil-fired power plant, respectively. The contamination indices also supported the finding that light lanthanoids were the major REE elements contaminating the industrial and traffic area and to some extent the dumpsite and residential area. Because heavy contamination was experienced in the industrial and traffic area, and to some extent the dumpsite area, the calculated contamination factor results were attributed to fossil fuel emissions from industrial and vehicular activities. This assertion was also supported by the degree of contamination, which showed severe contamination in two areas (industrial and traffic area). Meanwhile, the very high degree of contamination in those areas was a result of the domination of light lanthoid elements in these areas. Similarly, the high pollution index observed in the industrial and traffic area also attest to the fact that light lanthanoids were the major polluting elements of REEs in the study area.

### Limitations

We were unable to analyze for Pm due to the inability of the equipment used for REE analysis to detect Pm.

## Conclusions

The present study reported the findings of the investigation of REE concentrations of atmospheric dusts within the study area, in order to determine their sources and possible level of pollution. It was observed that the REEs in atmospheric dust were mainly contributed from natural source materials such as soil and weathered rocks. This was established by the close similarity of REE and ΣREE concentrations of the atmospheric dust geology of the study area and the similarity of chondrite-normalized REE patterns of both atmospheric dust and the local soil and rock of the study area. Several chondrite-normalized REE ratios such as La*_N_*/Yb*_N_*, La*_N_*/Sm_N_, and Gd_N_/Yb*_N_*_,_ LREE/HREE also showed that most of the atmospheric dusts were primarily sourced from the weathering of the parent rocks and from local soil of the study area. However, slight deviations from the conventional pattern of natural sources were observed for dusts of the industrial and traffic, and to some extent the dumpsite area. These dusts were anthropogenically introduced from the oil-fired power plant and vehicular exhaust as established from their higher REE and ΣREE concentrations compared to their values in the local soils in some samples. The higher REE ratio fractions and similarity to different established standards used in the determination of anthropogenic input also supports this assertion. The contamination factor, degree of contamination and pollution index calculated attest to the fact that the light lanthanoid elements of REE were the major polluting elements in the atmospheric dust of the industrial and traffic areas and to some extent the dumpsite area. The residential area was slightly contaminated by these elements, while the atmospheric dust of the remote area mainly reflected the background values and indicated geogenic contribution. The level of pollution of REEs in the industrial and traffic areas should be monitored and regulated to prevent further increases.
